# Body Fat Percentage in Relation to Lung Function in Individuals with Normal Weight Obesity

**DOI:** 10.1038/s41598-019-38804-3

**Published:** 2019-06-20

**Authors:** Yuan-Yuei Chen, Tung-Wei Kao, Wen-Hui Fang, Chung-Ching Wang, Yaw-Wen Chang, Hui-Fang Yang, Chen-Jung Wu, Yu-Shan Sun, Wei-Liang Chen

**Affiliations:** 1 Department of Internal Medicine, Tri-Service General Hospital Songshan Branch; and School of Medicine, National Defense Medical Center, Taipei, Taiwan Republic of China; 2 Division of Family Medicine, Department of Family and Community Medicine, Tri-Service General Hospital; and School of Medicine, National Defense Medical Center, Taipei, Taiwan Republic of China; 3 Health Management Center, Department of Family and Community Medicine, Tri-Service General Hospital; and School of Medicine, National Defense Medical Center, Taipei, Taiwan Republic of China; 4 Division of Geriatric Medicine, Department of Family and Community Medicine, Tri-Service General Hospital; and School of Medicine, National Defense Medical Center, Taipei, Taiwan Republic of China; 5 Graduate Institute of Clinical Medical, College of Medicine, National Taiwan University, Taipei, Taiwan Republic of China; 6 Division of Family Medicine, Department of Community Medicine, Taoyuan Armed Forces General Hospital, Taoyuan, Taiwan Republic of China

**Keywords:** Obesity, Epidemiology

## Abstract

Accumulating evidence indicates the association between obesity and lung function. However, no previous study has examined whether obesity affects lung function in normal weight participants with high body fat. We hypothesized that subjects with normal weight obesity (NWO) were inversely associated with lung function in Taiwan. The study sample was composed of participants who attended health examinations at the Tri-Service General Hospital from 2010 to 2016. A total of 7801 eligible participants who were classified as NWO were divided into quartiles by percentage body fat (PBF), which was measured by bioelectrical impedance analysis (BIA). A multivariable linear regression was performed to assess the association between PBF quartiles and pulmonary function. The relationship between PBF and the presence of obstructive and restrictive lung diseases was analyzed by a logistic regression. PBF quartiles were closely associated with reduced forced expiratory volume in one second (FEV1) and forced vital capacity (FVC) in all adjusted models. This relationship remained significant in the male population, and a dose-dependent effect was observed. Increased PBF was associated with increased risks for the presence of restrictive lung diseases. These results presented a novel finding that body fat exhibited an inverse association with pulmonary function in NWO subjects. More comprehensive management of subjects with normal weight but high body fat, which might contribute to metabolic dysfunction and impaired pulmonary function, is needed.

## Introduction

The prevalence of obesity is progressively increasing worldwide and is becoming a major public health problem in Taiwan^1,2^. Obesity is associated with high risks of insulin resistance^3^, metabolic syndrome^4^, and cardiometabolic diseases^5^, The adverse effect of overweight and obesity on reduced lung function have been observed in numerous studies^6,7^. Body mass index (BMI) was inversely associated with lung function and lung volume^8–10^. In a longitudinal analysis, increased BMI in people who had a BMI ≥ 26.4 kg/m^2^ was associated with impaired lung function in young healthy adults^11^. Overweight is potentially associated with decreased forced expiratory volume in one second (FEV1)^12–14^. An inverse relationship between obesity and forced vital capacity (FVC) was also reported in previous studies^15,16^. The common definition for adiposity or obesity relies on BMI, which is simple and reasonable^17^. However, increased body fat and elevated lean mass are rarely distinguished, particularly in subjects with BMI >30 kg/m^2^^18,19^.

Normal weight obesity (NWO), an emerging viewpoint initially described by De Lorenzo *et al*., represents the association between normal weight and high body fat with metabolic dysfunction^20^. Previous evidence indicates that excess adiposity in NWO individuals might be associated with cardiometabolic disorders, metabolic syndrome, and cardiovascular risk factors^21^. However, no previous study has examined whether obesity influences lung function in an NWO population. The objective of the present study was to investigate the association between body fat percentage (PBF) with lung function measured by pulmonary function tests (PFT) in a cross-sectional study composed of participants who attended health examinations at the Tri-Service General Hospital (TSGH) in Taiwan.

## Results

### Characteristics in participants with a normal BMI based on PBF quartiles

The characteristics of 7801 eligible subjects divided by PBF quartiles are provided in Table 1. The mean age was Q1: 42.31 ± 12.74 years, Q2: 46.20 ± 12.40, Q3: 46.07 ± 11.99 and Q4: 49.21 ± 13.20, respectively. Participants in higher PBF quartiles exhibited reduced FEV1 and FVC. Demographic factors such as age, height, waist circumference and biochemical data and medical history exhibited significant differences across these PBF quartiles

**Table 1:** Characteristics of study sample in normal weight obesity.

Variables	Q1 PBF < 21 (N = 1952)	Q2 21 < PBF < 26 (N = 1967)	Q3 26 < PBF < 31 (N = 1950)	Q4 PBF > 31 (N = 1932)	P-value
**Continuous Variables**, **mean (SD)**					
FEV1 (L)	3.51 (0.75)	2.97 (0.71)	2.57 (0.58)	2.32 (0.54)	<0.001
FVC (L)	4.08 (0.84)	3.47 (0.81)	2.99 (0.67)	2.70 (0.61)	<0.001
FEV1/FVC	0.99 (0.11)	0.99 (0.12)	0.99 (0.11)	1.00 (0.12)	<0.001
Age (years)	42.31 (12.74)	46.20 (12.40)	46.07 (11.99)	49.21 (13.20)	<0.001
Height (cm)	171.57 (7.28)	166.36 (7.76)	160.91 (6.85)	157.99 (6.20)	<0.001
WC (cm)	77.89 (6.03)	77.90 (7.44)	75.93 (6.87)	77.71 (6.42)	<0.001
Lean mass (kg)	29.59 (3.91)	25.53 (4.99)	21.52 (3.39)	19.56 (2.96)	<0.001
TG (mg/dL)	99.70 (57.31)	108.42 (66.91)	100.50 (75.27)	107.33 (85.58)	<0.001
HDL-C (mg/dL)	54.62 (13.52)	54.94 (14.37)	59.86 (15.09)	59.20 (14.02)	<0.001
FPG (mg/dL)	89.55 (14.82)	92.20 (20.87)	90.62 (17.99)	92.70 (18.14)	<0.001
UA (mg/dL)	5.91 (1.22)	5.49 (1.44)	4.80 (1.19)	4.82 (1.06)	<0.001
TSH (IU/mL)	2.15 (1.45)	2.26 (1.48)	2.28 (1.51)	2.56 (2.03)	<0.001
**Category Variables**, **(%)**					
Gender (male)	1518 (91.7)	1020 (59.8)	292 (17.1)	40 (2.3)	<0.001
Smoking	768 (39.4)	603 (30.7)	313 (16.1)	155 (8.1)	<0.001
Drinking	1027 (59.5)	860 (49.3)	607 (35.0)	454 (25.8)	<0.001

FEV1, forced expiratory volume in one second; FVC, forced vital capacity; WC, waist circumference; TG, triglyceride; HDL-C, high-density lipoprotein cholesterol; FPG, fasting plasma glucose; UA, uric acid; TSH, thyroid stimulating hormone.

### Pulmonary function in participants with normal BMI based on PBF quartiles

In continuous models (Table 2), PBF quartiles were closely associated with reduced FVC and FEV1 in all adjusted models: FEV1: Q2 = −0.115 (95% CI = −0.156, −0.074), Q3 = −0.159 (95% CI = −0.209, −0.110), Q4 = −0.164 (95% CI = −0.221, −0.107); FVC: Q2 = −0.118 (95% CI = −0.166, −0.070), Q3 = −0.179 (95% CI = −0.237, −0.121), Q4 = −0.189 (95% CI = −0.256, −0.123). However, no significant association was observed for FEV1/FVC.

**Table 2:** Association between PBF and lean mass in quartile and pulmonary function in NWO population.

Variable	Quartile	Model^a^ 1 β^b^ (95% CI)	*P* Value	Model^a^ 2 β^b^ (95% CI)	*P* Value	Model^a^ 3 β^b^ (95% CI)	*P* Value
	PBF						
FEV1	Q2 v.s. Q1	−0.142 (−0.182, −0.102)	<0.001	−0.129 (−0.170, −0.089)	<0.001	−0.115 (−0.156, −0.074)	<0.001
	Q3 v.s. Q1	−0.191 (−0.238, −0.143)	<0.001	−0.181 (−0.229, −0.134)	<0.001	−0.159 (−0.209, −0.110)	<0.001
	Q4 v.s. Q1	−0.201 (−0.253, −0.148)	<0.001	−0.201 (−0.253, −0.148)	<0.001	−0.164 (−0.221, −0.107)	<0.001
FVC	Q2 v.s. Q1	−0.149 (−0.195, −0.103)	<0.001	−0.139 (−0.185, −0.092)	<0.001	−0.118 (−0.166, −0.070)	<0.001
	Q3 v.s. Q1	−0.219 (−0.275, −0.164)	<0.001	−0.212 (−0.267, −0.156)	<0.001	−0.179 (−0.237, −0.121)	<0.001
	Q4 v.s. Q1	−0.243 (−0.304, −0.182)	<0.001	−0.243 (−0.304, −0.182)	<0.001	−0.189 (−0.256, −0.123)	<0.001
FEV1/FVC	Q2 v.s. Q1	−0.003 (−0.010, 0.004)	0.380	−0.003 (−0.010, 0.004)	0.379	−0.005 (−0.012, 0.002)	0.180
	Q3 v.s. Q1	−0.001 (−0.009, 0.007)	0.762	−0.001 (−0.009, 0.007)	0.758	−0.004 (−0.013, 0.004)	0.328
	Q4 v.s. Q1	0.002 (−0.007, 0.011)	0.732	0.002 (−0.007, 0.011)	0.732	−0.003 (−0.013, 0.007)	0.547

^a^Adjusted covariates:

Model 1 = age, gender, height.

Model 2 = Model 1 + age*height.

Model 3 = Model 2 + WC, TG, HDL-C, FPG, UA, TSH + history of smoking.

^b^β coefficients is interpreted as change of PBF in different pulmonary function index.

The association between PBF and pulmonary function in based on gender differences is presented in Table 3. The relationship remained significant in the male population and a dose-dependent effect was observed. Specially, higher PBF quartiles exhibited lower pulmonary function in the fully-adjusted model: FEV1: Q2 = −0.101 (95% CI = −0.156, −0.047), Q3 = −0.169 (95% CI = −0.253, −0.085), Q4 = −0.306 (95% CI = −0.510, −0.102); FVC: Q2 = −0.088 (95% CI = −0.151, −0.025), Q3 = −0.177 (95% CI = −0.273, −0.081), Q4 = −0.312 (95% CI = −0.547, −0.078). By contrast, only the highest PBF quartile exhibited significant associations with reduced FVC in the female population after fully adjusting for covariables (**β = **−0.124, 95% CI = −0.230, −0.019).

**Table 3:** Association between PBF in quartile and pulmonary function tests in NWO population in gender difference.

Gender	Variable	Quartile	Model^a^ 1 β^b^ (95% CI)	*P* Value	Model^a^ 2 β^b^ (95% CI)	*P* Value	Model^a^ 3 β^b^ (95% CI)	*P* Value
		PBF						
Male	FEV1	Q2 v.s. Q1	−0.118 (−0.169, −0.067)	<0.001	−0.119 (−0.170, −0.067)	<0.001	−0.101 (−0.156, −0.047)	<0.001
		Q3 v.s. Q1	−0.197 (−0.275, −0.118)	<0.001	−0.196 (−0.274, −0.118)	<0.001	−0.169 (−0.253, −0.085)	<0.001
		Q4 v.s. Q1	−0.347 (−0.547, −0.146)	<0.001	−0.344 (−0.544, −0.143)	<0.001	−0.306 (−0.510, −0.102)	0.003
	FVC	Q2 v.s. Q1	−0.118 (−0.177, −0.059)	<0.001	−0.118 (−0.178, −0.058)	<0.001	−0.088 (−0.151, −0.025)	0.006
		Q3 v.s. Q1	−0.229 (−0.319, −0.138)	<0.001	−0.228 (−0.318, −0.138)	<0.001	−0.177 (−0.273, −0.081)	<0.001
		Q4 v.s. Q1	−0.381 (−0.611, −0.150)	<0.001	−0.378 (−0.609, −0.147)	<0.001	−0.310 (−0.545, −0.076)	0.010
	FEV1/FVC	Q2 v.s. Q1	−0.004 (−0.012, 0.004)	0.303	−0.005 (−0.012, 0.003)	0.257	−0.008 (−0.017, 0.000)	0.060
		Q3 v.s. Q1	−0.003 (−0.015, 0.009)	0.651	−0.002 (−0.015, 0.010)	0.685	−0.009 (−0.022, 0.004)	0.182
		Q4 v.s. Q1	−0.020 (−0.051, 0.011)	0.211	−0.018 (−0.049, 0.013)	0.249	−0.027 (−0.059, 0.004)	0.087
Female	FEV1	Q2 v.s. Q1	−0.014 (−0.105, 0.077)	0.761	−0.015 (−0.105, 0.076)	0.752	−0.011 (−0.101, 0.080)	0.817
		Q3 v.s. Q1	−0.061 (−0.148, 0.026)	0.169	−0.062 (−0.149, 0.025)	0.162	−0.047 (−0.134, 0.040)	0.289
		Q4 v.s. Q1	−0.097 (−0.184, −0.011)	0.027	−0.097 (−0.184, −0.011)	0.028	−0.063 (−0.152, 0.025)	0.161
	FVC	Q2 v.s. Q1	−0.054 (−0.162, 0.054)	0.328	−0.055 (−0.163, 0.053)	0.316	−0.049 (−0.157, 0.059)	0.372
		Q3 v.s. Q1	−0.114 (−0.217, −0.011)	0.031	−0.116 (−0.219, −0.013)	0.028	−0.097 (−0.201, 0.006)	0.066
		Q4 v.s. Q1	−0.165 (−0.268, −0.062)	0.002	−0.165 (−0.268, −0.062)	0.002	−0.124 (−0230, −0.019)	0.021
	FEV1/FVC	Q2 v.s. Q1	0.010 (−0.008, 0.028)	0.263	0.010 (−0.007, 0.028)	0.258	0.010 (−0.008, 0.027)	0.290
		Q3 v.s. Q1	0.011 (−0.006, 0.028)	0.192	0.011 (−0.005, 0.028)	0.186	0.010 (−0.007, 0.027)	0.234
		Q4 v.s. Q1	0.014 (−0.003, 0.031)	0.104	0.014 (−0.003, 0.031)	0.104	0.012 (−0.005, 0.030)	0.154

^a^Adjusted covariates:

Model 1 = age, gender, height.

Model 2 = Model 1 + age*height.

Model 3 = Model 2 + WC, TG, HDL-C, FPG, UA, TSH + history of smoking.

^b^β coefficients is interpreted as change of PBF in different pulmonary function index.

### Association between PBF and the presence of obstructive and restrictive lung diseases in the NWO population

In Table 4, PBF was significantly associated with the increased risk of restrictive lung diseases in the NWO population with odd ratios (ORs) of 1.029 (95% CI = 1.007–1.050) in the fully-adjusted model. Taking gender into account (Table 5), PBF was associated with increased risk of both obstructive and restrictive lung disease in males after fully adjusting covariables with ORs of 1.112 (95% CI = 1.001–1.236) and 1.042 (95% CI = 1.009–1.076), respectively. However, no significant difference was found in female participants.

**Table 4:** Association between PBF and the presence of obstructive and restrictive lung defects in NWO population.

Outcomes	Model^a^ 1 OR^b^ (95% CI)	*P* Value	Model^a^ 2 OR^b^ (95% CI)	*P* Value	Model^a^ 3 OR^b^ (95% CI)	*P* Value
	PBF					
Obstructive	1.022 (0.957–1.092)	0.518	1.022 (0.956–1.092)	0.521	1.044 (0.969–1.125)	0.256
Restrictive	1.045 (1.026–1.065)	<0.001	1.046 (1.026–1.065)	<0.001	1.029 (1.007–1.050)	0.008

^a^Adjusted covariates:

Model 1 = age, gender, height.

Model 2 = Model 1 + age*height.

Model 3 = Model 2 + WC, TG, HDL-C, FPG, UA, TSH + history of smoking.

^b^OR is interpreted as change of PBF in different pulmonary diseases.

**Table 5:** Association between PBF and the presence of obstructive and restrictive lung diseases in NWO population in gender difference.

Gender	Outcomes	Model^a^ 1 OR^b^ (95% CI)	*P* Value	Model^a^ 2 OR^b^ (95% CI)	*P* Value	Model^a^ 3 OR^b^ (95% CI)	*P* Value
		PBF					
Male	Obstructive	1.046 (0.946–1.157)	0.377	1.054 (0.952–1.167)	0.314	1.112 (1.001–1.236)	0.049
	Restrictive	1.058 (1.029–1.088)	<0.001	1.059 (1.030–1.088)	<0.001	1.042 (1.009–1.076)	0.011
Female	Obstructive	1.003 (0.921–1.091)	0.952	1.004 (0.922–1.094)	0.925	1.001 (0.908–1.104)	0.981
	Restrictive	1.034 (1.008–1.061)	0.010	1.033 (1.007–1.059)	0.014	1.016 (0.988–1.045)	0.268

^a^Adjusted covariates:

Model 1 = age, gender, height.

Model 2 = Model 1 + age*height.

Model 3 = Model 2 + WC, TG, HDL-C, FPG, UA, TSH + history of smoking.

^b^OR is interpreted as change of PBF in different pulmonary diseases.

## Discussion

In our cross-sectional study, increased body fat exhibited a detrimental correlation with pulmonary function in an NWO population. Higher PBF quartiles were significantly associated with lower FEV1 and FVC, especially in males. Increased PBF was associated with increased risks for the presence of restrictive lung disease. To the best of our knowledge, our study was the first to examine the relationship between PBF and pulmonary function in NWO participants.

Accumulating evidence indicates that the association between obesity and both lung volume and lung function. Melo *et al*. demonstrated that reduced lung volume and capacity were present in obese subjects compared with healthy subjects^6^. BMI contributed to reduction in all lung volumes, especially the functional residual capacity^8^. In a study involving three large epidemiological datasets, severe obesity was a risk factor for recent asthma, and both FEV1 and FVC were significantly reduced with increasing BMI^22^. However, no previous study reported the interaction between lung function and individuals with NWO. The concept of NWO was defined as individuals with normal body weight and high PBF^23^. Emerging evidence indicates that the NWO population was significantly associated with increased risk of developing metabolic syndrome, cardiometabolic diseases and high mortality^21^. In a cross-sectional study of a representative sample of US adults, Chen *et al*. reported that increased number of metabolic syndrome components and individual components of metabolic syndrome exhibited a relationship with impaired FEV1 and FVC^24^. This finding strongly supported the viewpoint of our study and implied a plausible explanation for reduced lung function in the NWO population.

Lung function impairment is associated with increased PBF in obese individuals^25^. Similar results were presented in a study of elderly subjects, demonstrating that increased PBF had a negative impact on lung function in both genders^26^. These findings were consistent with our study findings suggesting that increased PBF was detrimental to lung function and related to several pulmonary diseases. Several potential mechanisms of the interaction between PBF and lung function are noted. Adiposity accumulation in the thoracic and abdominal regions might directly affect the downward movement of the diaphragm and chest wall properties^7^. Reduced lung volume resulted from the android pattern of body fat deposition by generating increased resistance to diaphragmatic contraction and impairing respiratory mechanics^27^. In addition to the mechanical pathway, the metabolic dysfunction caused by adipose tissue might contribute to the inverse effect. As the core pathophysiology of metabolic syndrome^3^, abdominal obesity was identified as the main factor by which increased C-reactive protein (CRP) concentrations caused by visceral fat accumulation^28^. Systemic inflammation might lead to reduced lung function given that CRP and interleukin-6 (IL-6) expressed in inflammatory lung epithelial cells^29^. Chronic obstructive pulmonary disease, a major cause of reduced FEV1, resulted from lung inflammation related to high serum levels of CRP^30,31^. Another plausible mechanism was that increased levels of cytokines, particularly IL-6, caused inflammatory cell infiltration into the pulmonary capillary endothelium and altered endothelial function^32^.

Although our study had the strengths of a large population-based survey and a novel finding regarding the impact of body composition on lung function in NWO individuals, the present analysis still has some potential limitations. First, causation between PBF and lung function was not determined due to the cross-sectional design. Long-term observations should be performed in future studies. Second, the study sample was obtained from a single medical center composed of Asian adult. Thus, the limited ethnic distribution of participants might not represent the effect of PBF on lung function in terms of racial differences. Finally, PBF was measured by BIA in the present study. Dual-energy X-ray absorptiometry (DEXA), which is the golden standard for body composition measurement internationally might be more accurate than BIA.

## Conclusion

The present study highlighted a novel finding that PBF was detrimentally correlated with lung function in an NWO population. The concept of NWO reflected the effect of metabolic dysregulation and systemic inflammation caused by increased PBF that might not detected by BMI. The relationship between lung function and the general definition of obesity based on weight and height has been challenged given that individuals with normal weight and high PBF exhibited the same results, and this effect might be more closely associated with the reduction of lung function. A better understanding and recognition of the complex interaction between body compositions and the pathophysiology of lung function should be addressed in the future studies.

## Methods

### Study design and participants selection

A cohort study involving participants aged 20 years old and older who attended health examinations at the TSGH from 2010 to 2016 was performed. Participants who were underweight (BMI < 18.5), overweight (24 < BMI < 27), and obese (BMI > 27) were excluded initially. Those with missing data such as biochemical examinations (n = 6182), body composition measurement (n = 21424), and PFT (n = 34519) were also excluded. The remaining 7801 participants were defined as an NWO population and divided into PBF quartiles: Q1 = PBF < 21, Q2 = 21 < PBF < 26, Q3 = 26 < PBF < 31, Q4 = PBF > 31. Study approval was obtained from the Institutional Review Board of TSGH, Taiwan. The TSGH IRB waived the informed consent because these data were analyzed anonymously. All methods were performed in accordance with the relevant guidelines and regulations of TSGH.

### Pulmonary function test and quality control

PFT measurements and results interpretation were defined by guidelines published by the European Respiratory and American Thoracic Societies^33–35^. Spirometry is a commonly used method for detecting lung function that represents a measure of volume against time. Subjects were requested to inhale forcefully and then to exhale vigorously for as long and as quickly as possible. Measurement of pulmonary mechanics assesses the ability of the lungs to move huge volumes of air quickly through the airways to identify airway obstruction. The entire study was continuously monitored by well-trained technicians, and a physician regularly supervised them and provided additional instructions. Furthermore, a senior physician reviewed the raw flow volume and volume-time curves and graded the subject’s performance. Data that were unacceptable as judged by a senior physician were excluded from the study. In the current study, the characteristics of PFT obtained from the study sample, including FEV1, FVC and the ratio of the two volumes (FEV1/FVC), were assessed in the analysis. The predicted values for FEV1 and FVC were calculated from the following equations obtained in a non-smoking and healthy Taiwanese adult sample^36^.

1. Predicted FEV1
  Men: 95.502-0.280 (Age)
  Women: 96.991-0.291(Age)
2. Predicted FVC

Men: -2.058 + 0.0456(Height)-0.0246(Age)

Women: -2.314 + 0.038(Height)-0.0173(Age)

### Definition of obstructive and restrictive lung diseases

Obstructive lung disease is a type of respiratory disease characterized by airway obstruction, including asthma, bronchiectasis and chronic obstructive pulmonary disease. Restrictive lung disease is a category of extrapulmonary, pleural, or parenchymal respiratory diseases that restrict lung expansion, leading to decreased lung volume and inadequate ventilation and/or oxygenation. Obstructive lung disease was identified as a FEV1/FVC <70% where FEV1 was reduced more than FVC. Restrictive lung disease was defined as a FEV1/FVC >70% where FVC was reduced more than FEV1^37^.

### Measurement of body composition

PBF was measured by BIA (InBody720, Biospace, Inc., Cerritos, CA, USA), an useful method for assessing body composition^38^. The BIA procedure was simple and noninvasive, and the results were reproducible and rapidly obtained.

### Covariates measurement

Information regarding cigarette smoking was obtained from participants by asking the following question: “How many packs do you smoke per day?”. BMI was estimated based on a general formula wherein the weight of the individual in kilograms was divided by the square of the height in meters (kg/m^2^). Biochemical data were collected by drawing blood samples from subjects after fasting for at least 8 hours and were measured by respective standard procedures.

### Statistical analysis

Statistical Package for the Social Sciences, version18.0 (SPSS Inc., Chicago, IL, USA) for Windows was employed for statistical estimations in the study. Student’s t test and Pearson’s chi-square test were used to examine the differences in terms of demographic information and biochemical data between PBF quartiles. The threshold for statistical significance was defined as a two-sided *p*-value of ≤0.05. Multivariable adjustment for related clinical variables was performed in an extend-model approach. Model 1: age, gender, and height; Model 2: Model l plus age x height; Model 3: Model 2 plus waist circumference (WC), triglyceride (TG), high density lipoprotein cholesterol (HDL-C), fasting plasma glucose (FPG), uric acid (UA), thyroid stimulating hormone (TSH), and history of smoking. Multivariable linear regression was applied to assess the association between PBF based on quartiles with pulmonary function parameters. Logistic regression was performed to assess the association between PBF and the presence of obstructive and restrictive lung diseases.
